# Influence Exerted upon the Teeth by the Silica Contained in Potable Waters

**Published:** 1858-01

**Authors:** 


### ARTICLE VIII.

*Influence exerted upon the Teeth by the Silica contained in Potable Waters.* By DR. GUILBERT. Translated from L'Art Dentaire, for the Am. Jour. Dental Science.

WE extract from an excellent thesis lately defended by M. Guilbert, physician at Noyeau, (*Etude sur les eaux potables,* thèse pour le doctorat soutenue a Paris le 23 juillet 1857, par M. Guilbert,) the considerations and the case which follow. We regret that M. Guilbert has not been able to prosecute further the investigations which he had commenced upon this interesting subject; but we hope he will not allow himself to be discouraged by obstacles such as he has encountered, and that he will continue the study of a hygienic question, which interests the population of a great number of localities in our country.

"Silica enters into the composition of bones; we find it in the ashes of tumors and of the various tissues of the economy. It is a substance contained in extremely minute proportions in the majority of the articles which constitute our food, where it exists in a form which increases its solubility and facilitates its assimilation. The same may be said of the silica contained in the water we drink; it is there in the liquid state, its solution being accomplished undoubtedly by the aid of alkali, either free or carbonated; it may be carried without modification, even into the nutritious vessels of the bones." (Sainte-Claire Deville, *Annales de chimie et de physique*, 3d series, vol. xxiii.) But if silica is useful in drinking water, may it not produce inconvenience when it is present in too great proportion?

The following observation will probably put us in the way of discovering certain facts.

We know how frequent are losses of teeth. They are especially common in the Noyonnais and throughout Picardy. If carelessness and negligence the most common cause, still, in certain persons, in spite of the greatest care, the teeth become carious, blacken, or become covered with a coating, which the daily use of the brush is insufficient to remove. The following case has been narrated to me.

M. J. has very white teeth, of which he is scrupulously careful. Before living in Ribécourt, he had never observed any deposit of tartar upon them. But, while he resided in that town, he was obliged, from time to time, to remove with an iron instrument, the concretions which formed upon his teeth. He then drank from a source which leaves a very abundant silicious deposit. Having had occasion to evaporate a certain quantity of this water, he treated it with hydrochloric acid, and there remained much silica in suspension. Having changed his residence and made no more use of this water, the deposit was not renewed. M. J. attributes the production of the coating which covered his teeth to the excess of silica contained in the water.

It may be asked, what does this observation prove? It leads to other observations, and the hygiene of the teeth under the double aspect of health and beauty, merits special study.

The loss of the teeth depends upon a variety of causes; but when such a disorder is endemic in a country, it is probable that there is a dominant cause, and this cause, public opinion, which is often correct, places in the water used for drink. I hoped to establish a sort of comparison between the nature of the water and the frequency of losses of teeth in the Noyonnais, following the statistics of the councils of revision; but, in making these researches, I did not reckon upon the negligence of persons who were charged to procure them for me, and I am obliged to postpone this examination.

